# Effect of Co-infection of Low Pathogenic Avian Influenza H9N2 Virus and Avian Pathogenic *E. coli* on H9N2-Vaccinated Commercial Broiler Chickens

**DOI:** 10.3389/fvets.2022.918440

**Published:** 2022-06-28

**Authors:** Sherif I. A. Mahmoud, Kamel A. Zyan, Mohamed M. Hamoud, Eman Khalifa, Shahin Dardir, Rabab Khalifa, Walid H. Kilany, Wael K. Elfeil

**Affiliations:** ^1^Department of Avian and Rabbit Diseases, Faculty of Veterinary Medicine, Benha University, Benha, Egypt; ^2^Department of Poultry and Rabbit Diseases, Faculty of Veterinary Medicine, Cairo University, Giza, Egypt; ^3^Department Microbiology, Faculty of Veterinary Medicine, Matrouh University, Matrouh, Egypt; ^4^Department Veterinary Care and Laboratories Department, Cairo Poultry Corporate, Giza, Egypt; ^5^Reference Laboratory for Veterinary Quality Control on Poultry Production, Animal Health Research Institute, Agriculture Research Center, Ministry of Agriculture, Cairo, Egypt; ^6^Department of Avian and Rabbit Department, Faculty of Veterinary Medicine, Suez Canal University, Ismailia, Egypt

**Keywords:** avian influenza, H9N2 AIV infection, *E. coli*, mixed infection, broiler - chicken, vaccine

## Abstract

In the last 40 years, low pathogenic avian influenza virus (LPAIV) subtype H9N2 has been endemic in most Middle Eastern countries and of course Egypt which is one of the biggest poultry producers in the middle east region. The major losses with the H9N2 virus infections come from complicated infections in commercial broiler chickens, especially *E. coli* infection. In this work, 2,36,345 Arbor acres broiler chickens from the same breeder flock were placed equally in four pens, where two pens were vaccinated against LPAIV of subtype H9N2 virus, and the other two pens served as non-vaccinated controls. All were placed on the same farm under the same management conditions. A total of twenty birds from each pen were moved to biosafety level−3 chicken isolators (BSL-3) on days 21 and 28 of life and challenged with LPAIV-H9N2 or *E. coli*. Seroconversion for H9N2 was evaluated before and after the challenge. The recorded results revealed a significant decrease in clinical manifestations and virus shedding in terms of titers of shedding virus and number of shedders in vaccinated compared to non-vaccinated chickens. In groups early infected with LPAIV-H9N2 virus either vaccinated or not vaccinated, there was no significant difference in clinical sickness or mortalities in both groups, but in late infection groups with H9N2 alone, non-vaccinated infected group showed significantly higher clinical sickness in comparison with infected vaccinated group but also without mortality. In groups co-infected with *E. coli* (I/M) and H9N2, it showed 100% mortalities either in vaccinated or non-vaccinated H9N2 groups and thus reflect the high pathogenicity of used *E. coli* isolates, whereas in groups co-infected with *E. coli* (per os to mimic the natural route of infection) and LPAIV-H9N2, mortality rates were significantly higher in non-vaccinated groups than those vaccinated with H9N2 vaccine (15 vs. 5%). In conclusion, the use of the LPAIV H9N2 vaccine has significantly impacted the health status, amount of virus shed, and mortality of challenged commercial broilers, as it can minimize the losses and risks after co-infection with *E. coli* (orally) and LPAIV-H9N2 virus under similar natural route of infection in commercial broilers.

## Introduction

Low pathogenic avian influenza virus (LPAIV) subtype H9N2 virus infection is an endemic disease in nearly all Middle Eastern countries including Egypt, Iran, Israel, Saudi Arabia, Jordan, Kuwait, Lebanon, and the United Arab Emirates (1). LPAIV-H9N2 viruses found in the Middle East are mostly of the G1 “Western” sub-lineage, with occasional isolation of Y439 lineage viruses, possibly originating from wild birds (2). Whenever LPAIV of subtype H9N2 virus prevalence was investigated in developing countries, by surveys and sampling, the virus was found frequently, particularly in live bird markets “LBMs.” LBMs are a major way of disease transmission and zoonotic infections (3). In Egypt, where LBMs are the main market for chicken consumers, the prevalence of LPAIV-H9N2 infections is about 10%. A degree of hyper-endemicity exists in all the previous countries, which is not the same for the other influenza virus subtypes such as H5N1 and H7 subtypes. This difference may be due to the nature of the LPAIV phenotype of the virus, allowing repeated re-infection of the same birds and the same flocks of layers and breeders chickens (with longer life span than broilers chickens). Silent spreading is frequently occurring between farms and backyards birds (2). Despite being detected by real-time reverse transcription polymerase chain reaction (RT-qPCR) in 2006, the first isolation of LPAIH9N2 in Egyptian birds' dates back to December 2010 (4). Serological surveillance done in February 2009 revealed the presence of antibodies against the LPAI H9N2 subtype in domestic poultry flocks (5). For the broiler industry In Egypt, the most common diseases that affect the flocks and causing severe economic losses are respiratory pathogens that act either singly or in combination with each other. Clinical signs caused by many poultry respiratory pathogens are similar and confusing (6). This includes avian influenza, Newcastle disease, and infectious bronchitis. All show a huge economic impact because of their ability to induce high mortality independently or in association with each other organisms (7, 8). Avian pathogenic *E. coli* (APEC) is the most common infectious pathogen of all poultry species, resulting in multiple diseases in commercial poultry flocks. The most common disease is colibacillosis, which results in severe economic losses (9). APEC virulence is related to the presence of multiple factors that help the pathogen in causing the disease. *E. coli* can cause significant necrosis to the host cells due to the various proteases, hydrogen peroxide, nitrous oxide, and the release of proinflammatory cytokines, inhibiting phagocytosis, and affecting the normal functions of B- and T-lymphocytes. The presence of *E. coli* may be a powerful predisposing factor for several viral and bacterial infections including LPAI H9N2 (10). No efficient vaccine has been declared for APEC and antibiotics have been used widely in poultry flocks for controlling this disease, leading to an extensive antimicrobial resistance (11). Infection of broiler chickens with *E.coli* before, after, or even during the infection with LPAI H9N2 induces severe clinical signs with high mortality; such two major pathogens can affect broiler chickens much more than each alone (12). Likewise, co-infections of LPAIV-H9N2 with other respiratory pathogens, such as infectious bronchitis virus (IBV), *Mycoplasma gallisepticum, Staphylococcus aureus, Escherichia coli, and Ornithobacterium rhinotracheale*, can exacerbate H9N2 infection, resulting in high morbidity and mortality (13). The co-infection of H9N2 virus and avian pathogenic *E.coli* potentiates the pathological picture of each other as the replication of the AIV-H9N2 virus leads to significant upregulation of some essential proteins associated with avian pathogenic *E.coli* adhesion (transforming growth factor beta-1, E-cadherin, fibromodulin, and so on.), innate immunity associated protein (beta-2-microglobulin, alpha-1-acid glycoprotein, TAP-binding protein, and so on.), and cell proliferation, differentiation and apoptosis (apoptotic protease-activating factor 1, mitogen-activated protein kinase, transforming growth factor beta-1, and so on.), and the upregulation processes enhance the bacterial pathogenicity and pathological effect, as a result of the pathological effect of the bacterial, it increases the level of protease-like enzymes in the respiratory and digestive tract tissues, which enhance the cleavability of LPAIV-H9N2 and the immunosuppressive effect of *E.coli* infection decrease the birds response to the virus infection, so the virus replicates much higher and potentiates its pathological picture (14–18). Also, co-infection of avian pathogenic *E.coli* and AIV-H9N2 virus can elevate the inflammatory mediators (TNF-α and INF-γ), and the immunosuppressive effect of *E.coli* infection decreases the birds response to the virus infection and potentiates the losses from the co-infection process (10, 18–20). The objective of this study was to evaluate the benefit of vaccinating broiler chickens with LPAIV H9N2 and the role of combined infection with both avian pathogenic *E. coli* (APEC) and LPAIV H9N2.

## Materials and Methods

### Ethical Approval

Animal studies were approved by the Animal Welfare and Research Ethics Committee of Benha University by approval no. BU2019421PX23, and all procedures were conducted strictly following the Guidelines for Care and Use of Laboratory Animals. Every effort was made to minimize animal suffering.

#### Birds and Vaccines

A total of 2,36,345 1-day-old Arbor Acres broiler chicks from vaccinated broiler breeders' flocks (1-day-old chicks with maternally derived immunity “MDA” against LPAIV-H9N2 virus) were obtained from the same breeder flock and placed in two closed broiler system farms in Elsaff, Giza Governorate. All were kept under commercial field conditions with proper biosecurity measures and received the same management standards. Farm A contained 117,170 birds and was vaccinated with MEFLUVAC H5+H9+ND7 combined vaccine “consist of H5N1 clade 2.2.1.2, H5N8 clade 2.3.4.4., NDV genotype-II, NDV-genotype-VII, and H9N2 inactivated vaccine seeds” (MEVAC Company, Egypt) at 10th day of age. Farm B was harbored 119,175 birds and vaccinated with MEFLUVAC H5+ND7 combined vaccine “containing H5N1 clade 2.2.1.2, H5N8 clade 2.3.4.4, NDV-genotype-II and NDV-Genotype-VII inactivated vaccine seeds” (MEVAC Company, Egypt) at 10th day of age. The birds also received vaccines for IBD (Univax BD “a live virus vaccine containing a mild strain (ST-12) of Infectious Bursal Disease,” MSD company, USA on day one of life then Bursine plus “Live freeze-dried intermediate plus IBD virus, Lukert strain,” Zoetis company, USA at day 13 of life), MEFLUVAC H5+ND7 combined vaccine (inactivated AIV-H5 vaccine (MEVAC Company, Egypt), and live bivalent ND-IB vaccine (Polimun ND Hitchner B1+IB H120, Biotest laboratory company at day one of life, Nobilis Clone 30+ IB Ma5 (MSD company at day 10 of life), Volvac LaSota (Boehringer-Ingelheim, Germany), Vaxsafe ND “ND V4 strain” (Bioproperties, Australia, at 16 day of life) as the part of routine vaccination program of commercial broilers in Egypt. All vaccines were used according to the manufacturer's recommendations.

Birds kept under field conditions underwent regular monitoring by RT-PCR on days 2, 4, 18, 21, 25, 27, and 31 of life for the detection of common circulating pathogens in the region, including AIV-H5, AIV-H9N2, NDV velogenic, IBV, IBD, avian nephritis virus, and chicken astrovirus using primer sets developed by Cairo Poultry Group diagnostic lab (house-made primers and probe, Unpublished data) and AgPath-ID™ One-Step RT-PCR Reagents Thermo Fisher Scientific Inc., Massachusetts, USA), according to the manufacturer's instructions (catalog number: 4387391, Thermo Fisher Scientific Inc., Massachusetts, USA) using Applied Biosystem 7500 RT-PCR engine, according to the manufacturer's instructions (Thermo Fisher Scientific Inc., Massachusetts, USA).

#### Experimental Design

The main objective of this work was to evaluate the effect of LPAIV-H9N2 vaccination (using inactivated vaccine) on the broiler chicks and response following challenge with LPAIV-H9N2 virus alone or combined with avian pathogenic E. coli under laboratory and commercial field conditions. The infection was applied in two stages, an early challenge on 21 days of life, 120 birds moved to BSL-3 (early challenge) and grouped as G-1-3(a-b), and for late challenge applied on day 28 of life, another 120 birds moved to BSL-3 and grouped as G-3-5(a-b) (late challenge), birds in G3a/b were challenged on day 21 of life with E. coli per os and kept under monitoring for 7 days (till 28 day of life) and challenged on day 28 of life with LPAIV-H9N2 via IN route to evaluate the effect of infection of H9N2 following E. coli infection. All birds kept in commercial farms from 1 day of life and moved to the BSL-3, 48 h before the challenge date, and every 12 h, cloacal and tracheal swabs were collected and checked with RT-PCR for AIV-matrix gene, NDV velogenic virus, and IBV using specific primer for each disease, to ensure that it is free from any infection before conducting the experimental infection on BSL-3 according to the experimental design.

#### Challenge Groups Under Laboratory Conditions

On the 19th day of life, 60 birds from each of Farms A and B were moved to BSL-3 isolators at the animal house of Mevac laboratories to conduct the challenge at 21st day of age (groups 1–3a/b, 20 birds each group) as described in Table 1 (challenge-1, early challenge). Group-1a from Farm A and G-1b from Farm B were challenged with H9N2 intranasally, and G-2a and G-2b were challenged with both H9N2 intranasally and *E. coli* intramuscularly. G-3a and G-3b were challenged with *E. coli* per the oropharyngeal route and then challenged 7 days later with H9N2 intranasally at 28^th^ day of age.

**Table 1:** Laboratory challenge groups, Groups:1a, 2a, 3, 4, 5a, and 6a (Challenge 1 at 21st day of life) and Groups 1b, 2b, 3, 4, 5b, and 6b (Challenge 2 at 28th day of life).

**G-No**.	**Challenge time**	**Birds No**.	**Vaccine**	**Challenge**	**Evaluation parameters**
G-1a	Early at 21^st^ of life	20	H5H9ND	H9N2; IN	•Serum, swabs before Challenge.
G-1b	Early at 21^st^ of life	20	H5ND	H9N2; IN	•Swabs for virus shedding at 3, 5, 7 DPC by RT-PCR
G-2a	Early at 21^st^ of life	20	H5H9ND	H9N2; IN+ E-Coli; IM	•Develop clinical signs and mortalities
G-2b	Early at 21^st^ of life	20	H5ND	H9N2; IN+ E-Coli; IM	•Serum samples at 10 DPC for challenge 2
G-3a	Early at 21^st&^ late at 28^th^ of life of life	20	H5H9ND	*E. coli* per os (at 21^st^ day of life) then H9N2; IN 7 days later (at 28^th^ day of life)	
G-3b	Early at 21^st&^ late at 28^th^ of life of life	20	H5ND	*E. coli* per os (at 21^st^ day of life) then H9N2; IN 7 days later (at 28^th^ day of life)	
G-4a	Late at 28^th^ of life	15	H5H9ND	H9N2; IN	
G-4b	Late at 28^th^ of life	15	H5ND	H9N2; IN	
G-5a	Late at 28^th^ of life	15	H5H9ND	H9N2; IN+ E-Coli; IM	
G-5b	Late at 28^th^ of life	15	H5ND	H9N2; IN+ E-Coli; IM	

*No, number of birds; IN, intranasal; IM, intramuscular; Per Os., oropharyngeal; G, group; DPC, days post-challenge; RT-PCR, reverse transcription polymerase chain reaction*.

On day 26th of life, 30 birds from each of Farms A and B were moved to BSL-3 isolators at the animal house of Mevac laboratories to conduct the challenge at 28th day of age as described in Table 1 (challenge-2, late challenge). Birds were divided into 4 different experimental groups; Group-4a from Farm A and G-4b were challenged with H9N2 intranasally, and G-5a and G-5b were challenged with H9N2 intranasally and *E. coli* intramuscularly. Birds in G-3a/b were previously challenged with *E. coli* per the oropharyngeal route in the first challenge “early challenge” (at 21st day of life) and kept under observation for 7 days and then challenged intranasally with LPAIV-H9N2 (at 28th day of life).

#### Hemagglutination Inhibition Test

Hemagglutination inhibition (HI) test was used to monitor the humoral immune response of each vaccine against the antigens of avian influenza H9N2, H5N1 (clade 2.2.1.1), H5N1 (clade 2.2.1.2), H5N8, and ND, which represents the circulating viruses in Egypt. HI test was performed according to the OIE manual (21, 22). Serial 2-fold serum dilutions in PBS were mixed with equal volumes (25 μl) of the virus-containing 4 hemagglutinating units (HAU), and then, 25 μl of washed chicken red blood cells was added. After incubation for 40 min at room temperature, HI titers were determined as reciprocals of the highest serum dilutions in which inhibition of hemagglutination was observed.

#### Challenge Virus

The LPAIV-H9N2 challenge was applied by the intranasal inoculation with 100 μl of allantoic fluid containing 6-log_10_ embryo infective dose 50 (EID^50^) of previously isolated and identified LPAI-H9N2 virus (A/chicken/Egypt/Elfeil-26/2017(H9N2), with GenBank accession number: MF620130, kindly provided by Dr. Wael Elfeil, Suez Canal University, Egypt (23, 24).

#### Challenge Bacteria

Avian pathogenic *E. coli “APEC"* (Poly3:O157-H7) was applied either by oral or intramuscular route with 100 μl of 10^6^ cfu/ml, this isolation previously evaluated its pathogenicity and showed 80% mortalities by IM injection in specific pathogen free chicks, and this bacterial isolate generously provided by Mevac bacteriology laboratory (25).

#### RT-QPCR for Virus Shedding

Tracheal and cloacal swabs were collected from the challenged birds for the detection of virus shedding by RT-PCR at 3, 5, and 7 days post-challenge, as per the OIE manual (22) using specific primers and probes as previously described (23); RT-qPCR titers were converted into log_10_ EID_50_/ml as described previously (26). Briefly, a triplicate of six 10-fold dilutions of challenge AIV-H9N2 (AIV-H9N2; 10^6^ EID_50_/ml) was used to generate a standard curve using stock virus dilutions from 10^1^ to 10^6^. Since PCR cycle threshold “(CT.)” is defined as the point at which the curve crosses the horizontal threshold line, virus log_10_ titers of a specimen were plotted against the CT value, and the best fit line was constructed. The linear range of the assay was from 1 to 10^6^ EID_50_/ml, with a correlation coefficient of 0.99. System detection limit was 0.5 EID_50_/ml as has been standardized and described previously (27). The AIV H9N2 titer in collected samples was derived by plotting the CT of an unknown against the standard curve and expressed in log_10_ EID_50_/ml equivalents.

#### Statistical Analysis

Whenever necessary, data were analyzed by the Student's *t*-test or by ANOVA followed by the application of Duncan's new multiple range test to determine the significance of differences between individual treatments and corresponding control (28).

## Results

### Serology Monitoring in Field Groups

#### AIV H9N2 Titers Monitoring in Field Groups and Pre-Challenge PCR Swabs

**Figure 1:**
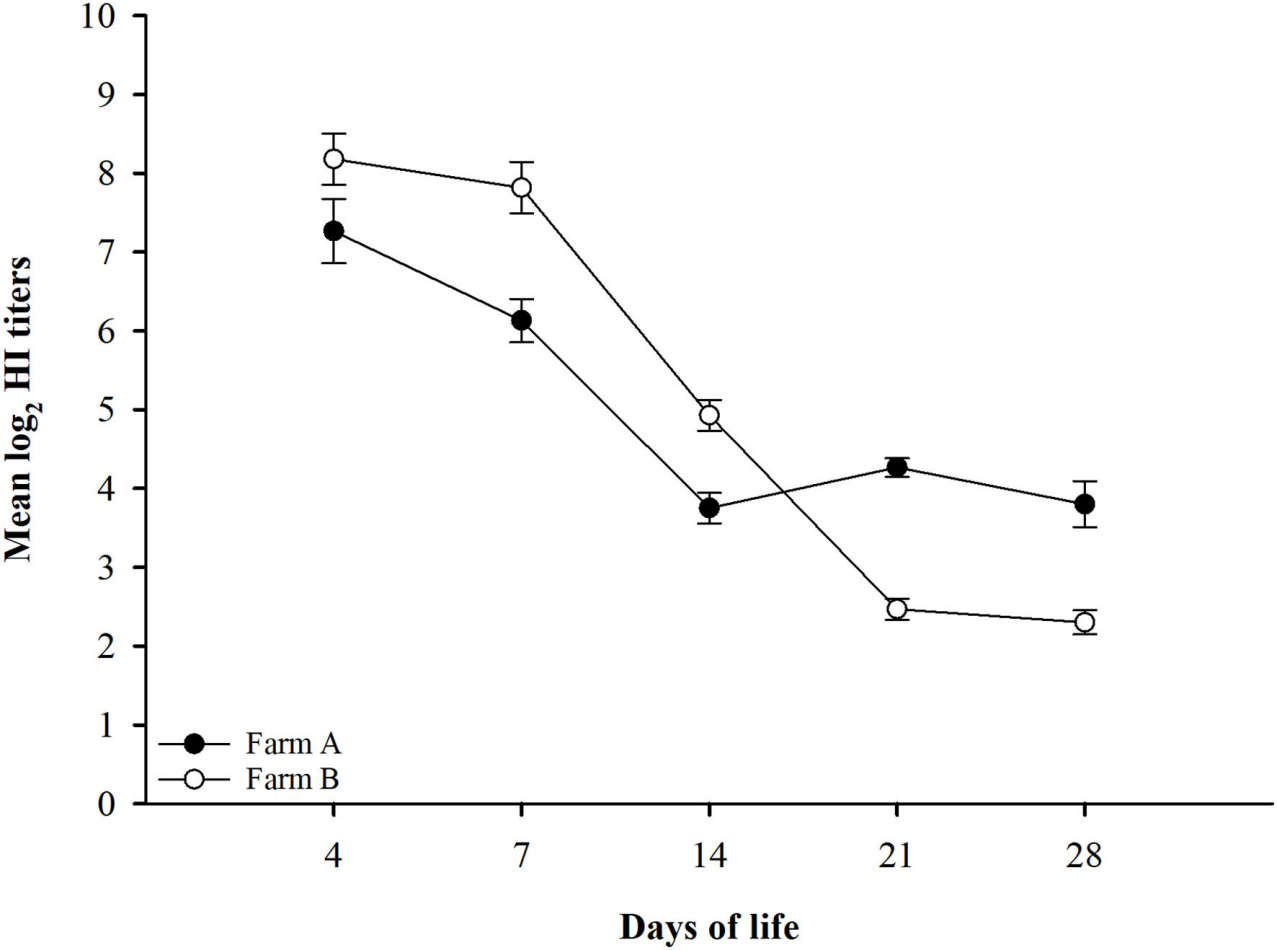
LPAIV-H9N2 HI test results graph with error bars for different field groups Error bars represent standard errors.

The findings from monitoring antibody titers in random serum samples collected from different farms at 4, 7, 14, 21, and 28 days of life for LPAIV-H9N2 are shown in Table 2 and Figure 1. There was a significant seroconversion in titers of H9N2-vaccinated group (Farm A) at 21 days of life GMT 4.3 compared to the non-vaccinated group (Farm B) GMT 2.5, and at 28 days of life GMT 3.8 compared to GMT 2.3 in the non-vaccinated group.

**Table 2:** AIV H9N2 HI GMT results for different field groups.

**Antigen**	**Age (Days)**	**Farm A**	**Farm B**
	**0 (MDA)**	**7.5 ±0.51**	**8.3 ±0.73**
AIV (H9N2)	4	7.3 ± 0.41	8.2 ± 0.33
	7	6.1 ± 0.27	7.8 ± 0.33
	14	3.8 ± 0.19	4.9 ± 0.20
	21	4.3 ± 0.12	2.5 ± 0.13
	28	3.8 ± 0.29	2.3 ± 0.15

*MDA, maternal derived antibodies, GMT, geometric mean titer, HI, hemagglutination inhibition assay*.

All swabs were collected upon the arrival of the birds to the BSL-3 (3 successive cloacal and tracheal swabs each 12 h), and the RT-PCR showed its negative with AIV-H5, AIV-H9N2, and velogenic NDV primers, which declare that the birds did not expose to infection before moving from farms or during transportation process from farm to BSL-3 units either groups moved on day 21 or 28 of life.

#### Monitoring of Other Disease Titers in Field Groups

**Figure 2:**
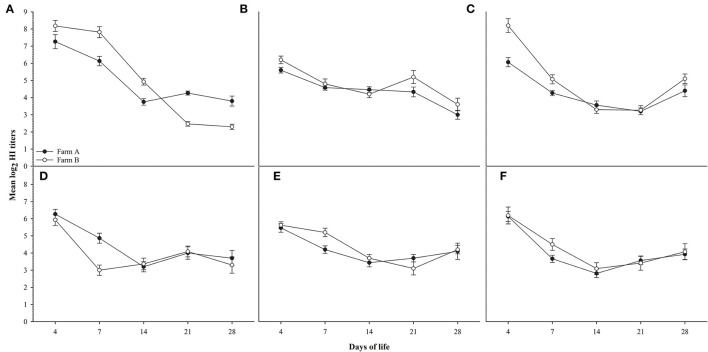
shows HI titers for ages 4,7,14,21 and 28 days for farms A&B, **(A)**: AIV H9N2, **(B)**: ND Lasota, **(C)**: ND Genotype VII, **(D)**: H5N1 clade 2.2.1.1, **(E)**: H5N1 clade 2.2.1.2, **(F)**: H5N8 clade 2.3.4.4. Error bars represent standard errors.

Random serum samples were collected from different farms at 4, 7, 14, 21, and 28 days of life for monitoring antibody titers for ND using LaSota, and ND Genotype VII antigens, AIV H5 antibodies against AIV (H5N1 clade 2.2.1.1), AIV (H5N1 clade 2.2.1.2), and AIV (H5N8 clade 2.3.4.4) antigens, and results did not show any significant difference between different field groups in titers for different antigens used compared to the significant difference observed in H9N2 titers as shown in Figure 2.

### Laboratory Group Challenge Results

#### Experiment-1: Early Challenge Protection Results

The results of the early challenge in different laboratory groups on the 21st day indicated the presence of clinical signs, clinical protection, mortality, and total protection as shown in percentages in Table 3.

**Table 3:** Experiment 1 (early challenge) protection results.

**Group**	**Clinical signs**	**Days post-challenge**							**Sick%**	**Clinical protection%**	**Mortality%**	**Protection%**
		**1**	**2**	**3**	**4**	**5**	**6**	**7**	**Sick/Total**	**Healthy/Total**	**Dead/Total**	**Alive/Total**
Gr.1a	Normal	20	20	20	20	20	20	20	0.0% 0/20	100.0% 20/20	0.0% 0/20	100.0% 20/20
	Sick	0	0	0	0	0	0	0				
	Dead	0	0	0	0	0	0	0				
Gr.1b	Normal	20	20	20	20	20	20	20	0.0% 0/20	100.0% 20/20	0.0% 0/20	100.0% 20/20
	Sick	0	0	0	0	0	0	0				
	Dead	0	0	0	0	0	0	0				
Gr.2a	Normal	0	0	0	0	0	0	0	100.0% 20/20	0.0% 0/20	100.0% 20/20	0.0% 0/20
	Sick	14	3	3	0	0	0	0				
	Dead	6	11	3	0	0	0	0				
Gr.2b	Normal	0	0	0	0	0	0	0	100.0% 20/20	0.0% 0/20	100.0% 20/20	0.0% 0/20
	Sick	15	3	2	0	0	0	0				
	Dead	5	12	3	0	0	0	0				
Gr.3a (early challenge)	Normal	20	20	20	20	20	19	18	15.0% 03/20	85.0% 17/20	0.0% 0/20	100.0% 20/20
	Sick	0	0	0	0	0	1	2				
	Dead	0	0	0	0	0	0	0				
Gr.3b (early challenge)	Normal	20	20	20	20	20	19	18	15.0% 03/20	85.0% 17/20	0.0% 0/20	100.0% 20/20
	Sick	0	0	0	0	0	1	2				
	Dead	0	0	0	0	0	0	0				

*Gr.1a, Group 1 (vaccinated + challenged with H9N2(IN)) No. = 20; Gr.1b, Group 5 (non-vaccinated + challenged with H9N2(IN)) No. = 20; Gr.2a, Group 2 (vaccinated + challenged with H9N2(IN) + E.coli(IM)) No. = 20; Gr.2b, Group 2b (non-vaccinated + challenged with H9N2(IN) + E.coli(IM)) No. = 20; Gr.3a, Group 3a (Vaccinated + challenged with E-Coli (Per Os.) then H9N2 7 days later(IN)) No. = 20; Gr.3b, Group 3b (non-vaccinated + challenged with E-Coli (Per Os.) then H9N2 7 days later(IN)) No. = 20. Sick birds mean birds showed lethargy, drop feather, nasal or ocular discharge and/ or respiratory manifestations. For birds in Gr3a/b the days post-challenge range 1–7 following to late challenge with H9N2 virus which equivalent to the first 7 out of17 days post-early challenge with APEC*.

#### Experiment-2: Late Challenge Results

The results of the challenge on the 28th day of different laboratory groups indicated the presence of clinical signs, clinical protection percentage, mortality percentage, and total protection percentage as shown in Table 4.

**Table 4:** Experiment-2 (late challenge) protection results.

**Group**	**Clinical signs**	**Days post-challenge**										**Sick%**	**Clinical protection%**	**Mortality%**	**Protection%**
		**1**	**2**	**3**	**4**	**5**	**6**	**7**	**8**	**9**	**10**	**Sick/Total**	**Healthy/Total**	**Dead/Total**	**Alive/Total**
Gr.4a	Normal	15	15	15	15	14	15	15	15	15	15	6.7% 1/15^*^	93.3% 14/15^*^	0.0% 0/15	100.0% 15/15
	Sick	0	0	0	0	1	0	0	0	0	0				
	Dead	0	0	0	0	0	0	0	0	0	0				
Gr.4b	Normal	15	10	9	9	12	15	15	15	15	15	40.0% 6/15^*^	60.0% 9/15^*^	0.0% 0/15	100.0% 15/15
	Sick	0	5	6	6	3	0	0	0	0	0				
	Dead	0	0	0	0	0	0	0	0	0	0				
Gr.5a	Normal	0	0	0	0	0	0	0	0	0	0	100.0% 15/15	0% 0/15	100.0% 15/15	0.0% 0/15
	Sick	15	13	10	7	6	0	0	0	0	0				
	Dead	0	2	3	3	1	6	0	0	0	0				
Gr.5b	Normal	0	0	0	0	0	0	0	0	0	0	100.0% 15/15	0.0% 0/15	100.0% 15/15	0.0% 0/15
	Sick	15	12	10	5	0	0	0	0	0	0				
	Dead	0	3	2	5	5	0	0	0	0	0				
Gr.3a (late Challenge)	Normal	18	18	18	17	17	17	16	16	19	19	15.0% 3/20^*^	85.0% 17/20^*^	5.0% 1/20^*^	95.0% 19/20^*^
	Sick	2	2	2	3	3	3	3	3	0	0				
	Dead	0	0	0	0	0	0	1	0	0	0				
Gr.3b (late challenge)	Normal	18	18	18	17	17	9	11	15	17	17	40.0% 8/20^*^	60.0% 12/20^*^	15.0% 3/20^*^	85.0% 17/20^*^
	Sick	2	2	2	3	3	8	6	2	0	0				
	Dead	0	0	0	0	0	3	0	0	0	0				

*Gr.4a, Group 4a (vaccinated + challenged with H9N2(IN)) No. = 15; Gr.5a, Group 5a (vaccinated + challenged with H9N2(IN)+ E. coli (IM)) No. = 15; Gr.3a, Group 3a (vaccinated + challenged with E-Coli (Per Os. At 21st day of age) then H9N2 (IN)at 28th day of age) No. =20; Gr.3b, Group 3b (non-vaccinated + challenged with E-Coli (Per Os. At 21st day of age) then H9N2 (IN)at 28th day of age) No. =20; Gr.4b, Group 4b (non-vaccinated + challenged with H9N2(IN)) No. = 15; Gr.5b, Group 5b (non-vaccinated + challenged with H9N2(IN)+ E. coli (IM)) No. = 15. Sick birds mean birds showed lethargy, drop feather, nasal or ocular discharge and/ or respiratory manifestations;. For birds in Gr3a/b the days post-challenge range 1–10 following to late challenge with H9N2 virus which equivalent to the second 10 out of 17 days post-early challenge with APEC*.

* *: indicate significance difference between groups (p < 0.05)*.

#### Virus Shedding Following Experiment 1/2 of Challenge

Shedding was evaluated at 3, 5, and 7 days post-challenge for different groups in early and late challenge experiments (21st and 28th day of life, respectively.) as shown in Table 5.

**Table 5:** Virus shedding at 3, 5, and 7 days post-challenge, Experiment-1/2.

**Group**	**Virus shedding EID_50_**		**Virus shedding EID_50_**		**Virus shedding EID_50_**	
	**3 days post-challenge**		**5 days post-challenge**		**7 days post-challenge**	
	**Trachea**	**Cloaca**	**Trachea**	**Cloaca**	**Trachea**	**Cloaca**
Gr.1a	3.5 ± 0.9	2.4 ± 1.2	3.5 ± 0.89	1.3 ± 0.94	1.07 ± 0.85	nd
Gr.1b	3.8 ± 0.17	0.7 ± 1.27	3.8 ± 0.31	1.7 ± 1.5	nd	1.7 ± 1.05
Gr.2a	4.2 ± 0.68	1.7 ± 1.66	nd	nd	nd	nd
Gr.2b	4.7 ± 0.81	2.6 ± 0.48	nd	nd	nd	nd
Gr.3a	2.3 ± 0.7	3.7 ± 0.48	1.04 ± 1.8	1.69 ± 0.69	nd	nd
Gr.3b	3.7 ± 0.15	2.5 ± 2.2	1.44 ± 1.25	1.88 ± 0.8	nd	nd
Gr.4a	2.4 ± 2.1	nd	nd		nd	nd
Gr.4b	1.68 ± 1.4	0.7 ± 1.27	nt	nd	nd	nd
Gr.5a	1.68 ± 1.4	0.7 ± 1.27	nd	nd	nd	nd
Gr.5b	2.9 ± 0.76	2.6 ± 0.48	nd	nd	nd	nd

*EID_50_, egg infectious dose50; No., Number of birds; IN, intranasal; IM, intramuscular; Per Os., oropharyngeal; Gr.1a, Group 1a (vaccinated + challenged with H9N2(IN)) No. = 20; Gr.1b, Group 1b (non-vaccinated + challenged with H9N2(IN)) No. = 20; Gr.2a, Group 2a (vaccinated + challenged with H9N2(IN) + E.coli(IM)) No. = 20; Gr.2b, Group 2b (non-vaccinated + challenged with H9N2(IN) + E.coli(IM)) No. = 20; Gr.3a, Group 3a (vaccinated + challenged with E-Coli (Per Os.) then H9N2 7 days later(IN)) No. =20; Gr.3b, Group 3b (non-vaccinated + challenged with E-Coli (Per Os.) then H9N2 7 days later(IN)) No. =20; Gr.4a, Group 4a (vaccinated + challenged with H9N2(IN)) No. = 15; Gr.4b, Group 4b (non-vaccinated + challenged with H9N2(IN)) No. = 15; Gr.5a, Group 5a (vaccinated + challenged with H9N2(IN) + E. coli (IM)) No. = 15; Gr.5b, Group 5b (non-vaccinated + challenged with H9N2(IN) + E. coli (IM)) No. = 15. nd: not detected*.

#### Serology Monitoring Following Experiment 2 Challenge

Serum samples were collected from different groups at 10 days post-challenge for monitoring antibody titers for AIV H9N2, ND using LaSota antigen, AIV-H5 using AIV (H5N1 clade: 2.2.1.1), AIV (H5N1 clade: 2.2.1.2), and AIV (H5N8 clade: 2.3.4.4) antigens as shown in Table 6.

**Table 6:** Serology results for different groups 10 days post-challenge, Experiment 2.

**GMT Log2 HI titer**				
	**Gr.3a**	**Gr.3b**	**Gr.4a**	**Gr.4b**
**Antigen**	**No. = 8**	**No. = 8**	**No. = 8**	**No. = 8**
AIV (H9N2)	10.13 ± 0.83	10 ± 0.76	10.8 ± 0.46	10 ± 0.93
ND (LaSota)	3 ± 0.93	4.13 ± 1.73	8.8 ± 0.71	3.13 ± 0.83
AIV (H5N1/a)	6.5 ± 0.53	4.88 ± 1.13	7.5 ± 0.53	6.5 ± 0.53
AIV (H5N1/b)	7.38 ± 0.52	6.38 ± 1.19	8.5 ± 0.53	6.75 ± 0.89
AIV (H5N8)	4.25 ± 1.04	3.75 ± 1.83	4.5 ± 0.76	4.25 ± 1.39

*AIV (H5N1/b), Avian influenza H5N1 clade 2.2.1.1 antigen; AIV (H5N1/b): avian influenza H5N1 clade 2.2.1.2 antigen; Gr.3a, Group 3a (Vaccinated + challenged with E-Coli (Per Os. At 21st day of age) then H9N2 (IN)at 28th day of age); Gr.3b, Group 3b (non-vaccinated + challenged with E-Coli (Per Os. At 21st day of age) then H9N2 (IN)at 28th day of age); Gr.4a, Group 5a (non-vaccinated + challenged with H9N2(IN)); Gr.4b, Group 5b (non-vaccinated + challenged with H9N2(IN)*.

#### Bacterial Isolation Following Experiment 1/2 Challenge

*E. coli* was isolated from groups 2a and 2b following the challenge at 21 days of life, *E. coli* was isolated from groups 3a/b and 5a/b following challenge at 28 days of life as shown in Table 7.

**Table 7:** Bacterial isolation post-challenge, Experiment-1/2.

**Group**	**Time of isolation**	**Isolate**
Gr.2a	18 h post-challenge	*E. coli*: O157-H7 (poly 3)
	1 DPC	*E. coli*: O157-H7 (poly 3)
	3 DPC	E. coli: O157-H7 (poly 3)
Gr.2b	1 DPC	*E. coli*: O157-H7 (poly 3)
	2 DPC	*E. coli*: O157-H7 (poly 3)
	3 DPC	*E. coli*: O157-H7 (poly 3)
Gr.3a	6 DPC	*E. coli*: O157-H7 (poly 3)
Gr.3b	6 DPC	*E. coli*: O157-H7 (poly 3)
Gr.5a	1 DPC	*E. coli*: O157-H7 (poly 3)
	3 DPC	*E. coli*: O157-H7 (poly 3)
Gr.5b	3 DPC	*E. coli*: O157-H7 (poly 3)
	3 DPC	*E. coli*: O157-H7 (poly 3)

*Gr.2a, Group 2a (vaccinated + challenged with H9N2(IN)+ E. coli (IM)) on day 21 of life; Gr.2b, Group 2b (non-vaccinated + challenged with H9N2(IN) + E. coli (IM)) on day 21 of life; Gr.3a, Group 3a (vaccinated + challenged with E-Coli (Per Os. At 21st day of age) then H9N2 (IN)at 28th day of age); Gr.3b, Group 3b (non-vaccinated + challenged with E-Coli (Per Os. At 21st day of age) then H9N2 (IN)at 28th day of age); Gr.5a, Group 5a (vaccinated + challenged with H9N2(IN)) at day 28 of life; Gr.5b, Group 5b (non-vaccinated + challenged with H9N2(IN) + E. coli (IM)) at day 28 of life*.

## Discussion

This study aimed to evaluate the role of vaccinating broiler chickens with inactivated LPAIV-H9N2 and the results of protection either with single H9N2 infection or as co-infection of both avian pathogenic *E. coli* (APEC) and LPAIV-H9N2. Co-infection of LPAIV-H9N2 (intranasal) with E-coli O157 (intramuscular injection) either on days 21 or 28 of life showed 100% mortalities in both vaccinated and non-vaccinated groups (2a/b and 5a/b) due to a septicemic reaction following the parenteral infection of the avian pathogenic *E.coli* (APEC), which is supported by the previous findings of El-Sawah et al. (29) and by Elfeil et al. (30) for the same bacterial isolates which is associated with 100% mortalities following I/M infection and following per os infection mortalities started after 7 days post-infection (25, 29). Per os infection of birds with *E. coli* O157 (as a natural route of infection) followed by intranasal infection with LPAIV-H9N2 7 days later (allowing sufficient time to produce infection) resulted in significantly higher clinical protection in vaccinated birds (G3a) than non-vaccinated birds (G3b) (85 vs. 60%, respectively), thus agreeing with the previous report of Wang et al. (15), who recorded the exacerbation of clinical signs in a mouse model co-infected with both AIV-H9N2 and *E. coli* (15) and report by Ma et al. (14), who reported the synergistic effect of LPAIV-H9N2 infection with avian pathogenic *E.coli* as the replication of H9N2 virus upregulated some essential proteins associated with the APEC pathogenicity and invasion-like (14). Infection with H9N2 virus 7 days after per os administration of *E. coli* (O157) showed relatively higher mortality (15%, 3/20) in non-vaccinated birds (G3b) compared to the birds received H9N2-inactivated vaccine (G3a) (5%, 1/20), and this difference may be associated with the damage of internal tissues and accumulated protease enzymes after *E. coli* infection, in addition to increase in the level of invasion and adherence protein transcription rate following H9N2 replication in intestinal and respiratory tissues, which potentiate the replication and pathological picture of APEC and thus increase the level of trypsin-like enzyme in the respiratory and GIT tissues, which later support and exaggerate the cleavability of H9N2 virus and intern the replication rate and thus may reflect the significant higher effect of the co-infection of H9N2 with APEC in non-vaccinated birds over-vaccinated (10, 14, 15, 31–33). There was no humoral immune response against H9N2 virus in non-vaccinated chicken, rendering the ability of virus transmission higher than that of vaccinated birds, and thus can explain the significant higher clinical manifestation in non-vaccinated infected birds either with H9N2 alone or co-infected with H9N2 and APEC. The protective effect of the humoral immune response associated with the inactivated H9N2 vaccine will decrease the load of H9N2 infection and limited its replication out the respiratory and GIT tissues and thus decrease the lead of the virus inside the birds tissue and reduced the associated inflammatory mediators (TNF-α and INF-γ); the immunosuppressive effect of E.coli infection decreases the birds response to the virus infection and potentiates the losses from the co-infection process (10, 15, 18–20, 23, 31–33). There is a significant higher level of mortalities and clinical sickness rates between group infected with APEC O:157 *via* I/M route over per os route, respectively, and thus associate with the nature of the bacteria as applying the infection *via* the IM route to ensure the on spot onset of septicemic infection and developing the bacteremia directly and thus can associated with 80–100% mortalities as previously described, while applying the vaccine *via* oral route needs more time to develop septicemia and may not develop it in all birds as we keeping commercial broilers and may exposed to *E.coli* during the first 21 day of live in the farm without clear clinical picture and developing systemic infection, and the 5% and 15% mortalities in H9N2-non-vaccinated and H9N2-vaccinated groups, respectively, agreed with the previous report by El-Sawah et al. (29), who reported that infection with APEC O:157 bacteria can associate with 5–25% mortalities in broiler chicks but needs 10–14 days following infection to give sufficient time to bacteria to adhere, colonize, and develop the systemic infections status (29), but the co-infection of APEC O:157 with H9N2 and the synergistic effect between them lead to develop the losses from day 7 post-infection in H92-non-vaccinated groups and reached to 15% mortalities by day 14 post-infection and 40% clinical sickness vs. 5% mortalities and 15% clinical sickness in H9N2-vaccinated groups in birds kept at BSL3 with negative pressure and filtrated air flow, which can explain in partial the higher losses in commercial farms due to the extra effect of in proper ventilation, over-crowdedness, co-infection with other pathogen or vaccine seed replications as previously described by Elfeil et al. (34), who highlighted that the application the avian influenza and NDV vaccines in farms can associate with around 10–15% lower protection level in comparison with the laboratory conditions (30, 34, 35). The multidrug-resistant *E. coli* is a serious problem facing the poultry industry as previously reported (36–38). The results from this trial may explain in part the exaggerated effect of LPAIV-H9N2 infection in commercial broiler farms in the Middle East region as the co-infection of LPAIV-H9N2 with the APEC work in a synergism and exaggerate the pathological picture for both pathogen, and the LPAIV-H9N2 circulating in the Middle east region still low pathogenic virus and losses associated with its infection in poultry farms is not due to the increased pathogenicity of the LPAIV-H9N2 virus, but rather to the heavy infection with multidrug-resistant *E. coli* and other pathogen such as IB, NDV, and IBD viruses in commercial broiler flocks (35, 37, 39, 40). This kind of synergy between different pathogens in broilers results in exaggerated clinical pictures, loss of weight, and higher mortalities. Birds in all groups either vaccinated with H5H9ND7 (inactivated H9N2- “vaccinated birds”) or H5ND7 (H9N2- “non-vaccinated birds”) vaccines showed similar seroconversion for H5 and ND, and only birds in field group A (vaccinated group) showed seroconversion for AIV-H9N2 on days 21 and 28 of life. Birds in field group B (non-vaccinated) did not show seroconversion for AIV-H9N2 on 28 days of life, indicating that the combination of three different antigens in one inactivated vaccine (like the trivalent H5H9ND in MEFLUVAC H5+H9+ND7) provided an immune response similar to the bivalent vaccine (MEFLUVAC H5+ND7) and declare that there is no negative effect of any vaccine antigens on the protection and evaluation parameters associated with the H9N2 vaccination, which agreed with the previous report about the safety and efficacy of both used vaccines in commercial broiler chicks with maternal derived antibodies (41). Use of a vaccine containing H9N2 at day 10 of life developed seroconversion for AIV-H9N2 at 28 days of life, in agreement with previous reports, and thus highlighted delay of the inactivated H9N2 vaccination in commercial broiler chicks to the 2nd week of life better than the 1 day of life application, especially in commercial broiler with maternal derived antibodies “birds came from vaccinated breeders” (23). Data obtained from the early challenge by AIV-H9N2 virus on day 21 of life, either in vaccinated or non-vaccinated groups, revealed no mortalities in both groups (G1a/b), which confirms the previous findings of Elfeil et al. (24), who reported that the AIV-H9N2 virus is of low pathogenicity and did not show clinical manifestation as a single pathogen in birds in the presence of humoral immune response (even remnants of maternally derived antibodies) (27) and thus may associated with the remnant of maternal derived immunity in the commercial broilers. On day 28 of life, the vaccinated group (G4a) showed significantly (*p* <0.05) better clinical protection against chicken sickness and developing clinical manifestations (93.3%) after the challenge compared to the non-vaccinated group (G4b, 60% protection against chicken sickness and developing clinical manifestations); this is in agreement with the previous report of Talat et al. (23), who reported that using inactivated H9N2 vaccine on day 7 of life in commercial broilers chicks with MDA with homologs and high concentrated antigen can provide protection over 90% protection against infection with AIV-H9N2 virus (23). The LPAIV-H9N2-inactivated vaccine will not completely solve the problem but may significantly improve the vitality, performance, and survival rates following the infection of commercial boilers with LPAIV-H9N2, especially in complicated cases such as persistent co-infection of APEC, which is very common case in the commercial poultry farms.

## Conclusions

Co-infection with LPAI-H9N2 and *E. coli*, especially the prolonged co-infection (over 7–14 days), may be the actual cause for the exaggerated losses associated with H9N2 infections in commercial broilers in endemic countries. The application of the LPAI-H9N2-inactivated vaccine strategy in commercial broilers may aid in controlling the complications associated with both LPAI-H9N2 and oral *E. coli* infections, by significant reduction the mortalities and clinical sickness. H9N2 vaccination should be associated with strict farm biosecurity measure to maintain superior clinical protection and minimize the bacterial co-infections especially with *E. coli*.

## Data Availability Statement

The raw data supporting the conclusions of this article will be made available by the authors, without undue reservation.

## Ethics Statement

The animal study was reviewed and approved by Animal Welfare and Research Ethics Committee of Benha University by approval No. BU2019421PX23.

## Author Contributions

SM, KZ, and WE: conceptualization. SM, EK, KZ, MH, and WE: methodology. SM, MH, KZ, and WE: software. SM, MH, and WE: validation, writing—original draft preparation, writing—review and editing, visualization, supervision, project administration, and funding acquisition. SM, EK, and WE: formal analysis and investigation resources. SM, MH, SD, RK, and WE: resources. SM, MH, EK, SD, and WE: data curation. All authors contributed efficiently in this work. All authors have read and agreed to the published version of the manuscript.

## Funding

This research was funded by the Benha University, grant number EGBEN2019421P27.

## Conflict of Interest

The authors declare that the research was conducted in the absence of any commercial or financial relationships that could be construed as a potential conflict of interest.

## Publisher's Note

All claims expressed in this article are solely those of the authors and do not necessarily represent those of their affiliated organizations, or those of the publisher, the editors and the reviewers. Any product that may be evaluated in this article, or claim that may be made by its manufacturer, is not guaranteed or endorsed by the publisher.
